# Development of a Hypertension Health Literacy Assessment Tool for use in primary healthcare clinics in South Africa, Gauteng

**DOI:** 10.4102/phcfm.v9i1.1305

**Published:** 2017-07-27

**Authors:** Nokuthula G. Mafutha, Sophie Mogotlane, Hester C. de Swardt

**Affiliations:** 1 Adelaide Tambo School of Nursing Science, Tshwane University of Technology, South Africa

## Abstract

**Background:**

Hypertension is a universal risk factor for cardiovascular morbidity and mortality in both the ageing and obese populations and patients must be literate in hypertension health issues to participate actively in the management of their disease. Little research has been done to investigate hypertension health literacy levels among South Africans.

**Aim:**

To develop a Hypertension Heath Literacy Assessment Tool to establish patients’ comprehension of the health education they receive in primary healthcare (PHC) clinics in Tshwane, Gauteng, South Africa.

**Setting:**

PHC clinics in Tshwane, Gauteng, South Africa.

**Methods:**

The design was quantitative, descriptive and contextual in nature. The study population comprised health promoters who were experts in the field of health, documents containing hypertension health education content and individuals with hypertension. Participants were conveniently and purposefully selected. A modified Delphi technique was used to develop and validate the Hypertension Health Literacy Assessment Tool (HHLAT). To ensure validity and reliability of the HHLAT, the tool was administered to 195 participants concurrently with the Learning Ability Battery (LAB).

**Results:**

There was a strong positive (*F* = 76.0, *p* < 0.0001, *R*^2^ = 28.25%) correlation between the LAB and the HHLAT. The HHLAT indicated that only 37 (19%) of the patients with hypertension had poor hypertension health literacy levels.

**Conclusion:**

The HHLAT is a valid tool that can be used in busy PHC clinics as it takes less than two minutes to administer. This tool can inform the healthcare worker on the depth of hypertension health education to be given to the patient, empowering the patient and saving time in PHC facilities.

## Introduction and background

Hypertension is a chronic, preventable non-communicable disease, the causes of which are related to genetics, behaviour and lifestyle.^1^ Although the pathology of hypertension is mainly located in the cardiovascular and nervous systems, many other factors tend to contribute to its cause.^1^ These include a diet high in salt and saturated fat, lack of exercise, obesity, excessive consumption of alcohol and cigarette smoking.^2^

According to the World Health Organization (WHO),^3^ the global prevalence of hypertension has escalated from 600 million to 1 billion between 1980 and 2008, and it is estimated to cause 7.5 million deaths annually. According to the WHO,^4^ the African region has a higher prevalence of hypertension than the American region. In 2011, prevalence of hypertension in the African region was reported to be 46%, while that for the American region was 35%. It is estimated that more than one-third of people in Africa have hypertension. Tibazwara and Damasceno^5^ state that 639 million people with hypertension live in developing countries with limited health resources, and according to these authors, people in these countries tend to have low awareness of hypertension and poor blood pressure control; the prevalence is also expected to increase by 2025. The poor control of hypertension might mean that hypertension will remain a major cause of disability-adjusted life years if measures are not taken to improve its control and management. Based on the Rapid Estimate Adult Literacy in Medicine – Revised (REALM-R) tool,^6^ the first author developed a tool to assess hypertension health literacy in PHC and to identify patients at risk of poor hypertension health literacy.

In order to manage and control hypertension, the patient has to comply with pharmacological and non-pharmacological therapies. Pharmacological therapies require a patient to adhere with medication,^7^ while non-pharmacological therapies require lifestyle modifications.^7,8^ Lifestyle modifications also require the patient to be able to read food labels and choose healthy food. Health literacy is the ability to read, write and comprehend the information related to health – in this case, specifically information related to hypertension management and control.^5^ This goes beyond simple reading ability. It involves the ability to understand instructions on prescriptions, appointment slips and health education pamphlets and the patients’ ability to make informed decisions concerning their own health.^6^

For patients to be health literate about hypertension, the healthcare professional needs to provide them with hypertension health education and to explain about hypertension, its management and prognosis.^9^ After this, the patients have the responsibility to understand and follow hypertension management regimens as prescribed to them for successful management and control of hypertension. A tool that specifically assesses the hypertension health literacy of patients before or after receiving hypertension health education was unavailable in South Africa at the time of the study. In 2010, Nkosi and Wright^10^ while investigating nutritional knowledge management practices of adults with hypertension in three primary healthcare (PHC) clinics in Tshwane discovered that participants were taught about hypertension in the clinics. However, none of the participants complied with the management therapy for hypertension, despite all of them confirming that they were given health education about hypertension management by a healthcare worker.^10^ This insight led to the realisation that there was a need for the development of a tool that would specifically assess hypertension health literacy.

Assessment tools for literacy and health literacy have been developed worldwide. Examples include the REALM-R.^6^ The REALM-R tool was designed to assess literacy in PHC. The words used in the tool are medical terms commonly used in PHC.^6^ The tool has 11 words ranging according to syllables. Its administration requires that the client reads the words in the tool out loud for the assessor. The first three words of the tool are not counted, only the following eight words are counted and scored. If a patient’s score is 6 or lower, the patient is identified as having poor health literacy.^6^ The first author used the principle of the REALM-R tool to develop a Hypertension Health Literacy Assessment Tool (HHLAT) that could be used to assess hypertension health literacy of individuals with hypertension.

Another assessment tool is the Learning Ability Battery (LAB) and is available in South Africa. The first author found it applicable for evaluating English literacy for patients at PHC clinics as English is the medium of instruction in South Africa. The LAB assessment tool is used to assess a person’s basic schooling grade and technical abilities, from grades 1 to 12 and also on the National Qualifications Framework levels 1–4. The tool has been acknowledged as being valid by the South African Qualification Authority.^11^ The tool consists of five sections, each comprising 10 English sentences. The five sections range in difficulty according to school grades.

Hypertension in South Africa affects about 6.3 million people, of whom about half are unaware that they have hypertension. Some people who know that they suffer from hypertension and are on medication have poorly controlled hypertension.^2^

Based on the principles of the REALM-R tool,^2^ the first author developed a tool to assess hypertension health literacy in PHC clinics to be able to identify patients who are at risk of poor hypertension health literacy. This tool will enable PHC practitioners to ensure that hypertension health education given to patients is provided according to the specific needs of the patient. For example, those at risk for poor hypertension health literacy will need more basic and simple, comprehensive and detailed hypertension health education compared to those who are not at risk.

The aim of the study was to develop a HHLAT that could be used in the PHC clinics of Tshwane, Gauteng, South Africa.

In order to develop the HHLAT, three phases were followed:

Phase 1: explore the oral (verbal) and existing printed health education content on prevention, management and control of hypertension.

Phase 2: based on the existing material, use the REALM-R tool as an approach to develop an HHLAT.

Phase 3: use the HHLAT to determine the hypertension health literacy level of patients with hypertension attending PHC clinics in Tshwane, Gauteng, South Africa. Also for validity purposes, correlate the HHLAT with the LAB.

## Research design and methods

### Research design

The design was quantitative, descriptive and contextual in nature.

### Population and sampling strategy

A statistician from Tshwane University of Technology was consulted and the statistician recommended the sample size as discussed here. For phase 1, the population comprised 12 health promoters and 50 pamphlets and posters. The health promoters were included in the study in order to record their hypertension health education to patients in the clinics.

For phase 2, the participants were a panel of 20 experts in health-related matters, and a validation panel of 50 experts in health-related matters. For phase 3, the participants consisted of 195 patients attending four PHC clinics. All participants were selected using non-probability convenience and purposive sampling as the participants were chosen based on eligibility criteria. The eligibility criteria for the panels of experts included the following: being experts in health-related matters such as hypertension, health education and health literacy, PHC, nursing, medicine, pharmacology and dietetics and having the willingness to give consent and to participate in the study. For the patients with hypertension, eligibility criteria were the ability to read English and give consent for participation, being 18 years of age and older and being managed for hypertension in the selected four PHC clinics.

### Research setting

The research context for phase 1 was 12 health promoters at 12 PHC clinics in Tshwane, Gauteng, South Africa. The first panel of 20 experts gathered in the same room at the Tshwane University of Technology. The validation panel of 50 experts validated the tool in the comfort of their own homes or offices as there was no need to meet. During the third phase, 195 patients were recruited from PHC clinics in the City of Tshwane, Gauteng, South Africa, to test the validated tool.

### Data collection

To collect the data for phases 1 and 2 of the study, a modified Delphi technique was followed.

## Data collection and analysis for phase 1

Round 1 of the modified Delphi technique involved the process of generating words, concepts and phrases.

Oral hypertension health education given by the health promoters in PHC clinics was recorded, transcribed and translated. The transcripts were labelled 1–12.

Themes for analysis were identified, guided by the literature regarding what hypertension health education content consisted of, for example, the definition of hypertension, risk factors, signs and symptoms, pharmacological management, non-pharmacological management and the complications of poorly controlled hypertension.

Printed health education materials (pamphlets and posters) were collected immediately after recording the health education. From the 50 printed hypertension health education materials, pamphlets and posters, duplicates were removed and only 11 remained. These were labelled 1–11.

From the 11 pamphlets and posters, themes were identified in terms of the following: definition of hypertension, risk factors, signs and symptoms, pharmacological management, non-pharmacological management and the complications of poorly controlled hypertension.

Each theme was allocated a specific colour for effective coding. Quantitative content analysis of the health promoters’ content and the content of the pamphlets and posters was done and these were analysed separately. In the analysis, transcript 1 was the point of departure for developing a frequency distribution list; for example, if the word ‘alcohol’ was highlighted in the transcript, it would be labelled ‘alcohol 1’ and when it appeared again in transcript 2 it would be labelled ‘alcohol 2’ (e.g. alcohol 1, 2), meaning that alcohol has appeared twice. This process was followed with the health education given by the health promoters and the printed hypertension health education materials.

The words, phrases and concepts generated from the process above were combined in order to develop one list of common words, phrases and concepts to be used by the first panel experts (Table 1). These were then arranged according to length in syllables, from one to six or more.

**TABLE 1:** Syllables and number of words, phrases and concepts from the printed and oral health education.

One syllable	Two syllables	Three syllables	Four syllables	Five syllables	Six or more syllables
Skip	High blood	Blood pressure	High blood pressure	Menopause women	Physical activity
Pill	Headaches	Tiredness	Hypertension	Avoid processed food	Don’t run out of medication
Take	Dizzy	Margarine	Vegetables	Stressful emotions	One teaspoon of salt a day
Drink	Reduce	Regular	Keep appointments	Comply with treatment	Avoid restaurant food
Keep	Chronic	Everyday	Incurable	Eat avocado	Exercise for 30 min
Stick	Chest pain	No muffins	Silent killer	High cholesterol	Poor eyesight (retinopathy)
Time	Treatment	No vetkoek	Shortness of breath	Normal blood pressure	Blocked arteries (coronary artery disease)
Legs	Better	Genetic	Check blood pressure	Pre-hypertension	Damaged internal organs
Eat	Feeling	No symptoms	No cigarettes	Reduce alcohol	Don’t borrow medication
Food	Little	Canola	No Aromat	Unhealthy diet	Take medication correctly
Weight	No salt	Tobacco	Obesity	Read ingredients	Can damaged internal organs
Salt	Tennis	History	Avoid fast food	-	More common in Africans
Spice	Change diet	Calorie	Lead to blindness	-	-
Add	Less starch	Less sugar	No bunny chow	-	-
Eat	Swelling	Heart failure	Know blood pressure	-	-
Oil	Less sweets	Poor eye sight	Brick margarine	-	-
Fast	Less beef	Lifestyle change	-	-	-
Boil	Less cheese	Read labels	-	-	-
Grill	Less spice	Overweight	-	-	-
Fry	Diet	Diabetes	-	-	-
Die	Do not	No fat cakes	-	-	-
Eye	Chicken	No stock cubes	-	-	-
Poor	Sodium	Stop smoking	-	-	-
Age	Walking	Heart attack	-	-	-
Stroke	Running	-	-	-	-
Death	Cleaning	-	-	-	-
Groups	Cooking	-	-	-	-
Herbs	Level	-	-	-	-
Beans	Support	-	-	-	-
-	Limit	-	-	-	-
-	Healthy	-	-	-	-
-	Spinach	-	-	-	-
-	Peanuts	-	-	-	-
-	Control	-	-	-	-
-	Intake	-	-	-	-
-	Ageing	-	-	-	-
-	Biltong	-	-	-	-
-	Tinned	-	-	-	-
-	Women	-	-	-	-
-	Drinking	-	-	-	-
-	Adding	-	-	-	-
-	Steam food	-	-	-	-
-	Avoid stress	-	-	-	-
-	Check-ups	-	-	-	-

## Data collection and analysis for phase 2

Round 2 of the modified Delphi technique involved identifying the most common hypertension words, phrases and concepts used in PHC to be used in the tool development.

Once the list of words was generated (Table 1), the first panel experts were invited to select the most commonly used words, phrases and concepts in hypertension health education from the list. The aim was for the experts to select the most appropriate and common words, phrases and concepts to be used in the development of the tool.

The list of words according to syllables, ranging from one syllable to six or more, was presented to the experts to vote on the most important items from the list of commonly used words, phrases and concepts in hypertension health education. Starting from one syllable, the researcher mentioned every word in the list and the experts voted by raising a hand if they thought the word, phrase or concept was important in hypertension health education.

To manage this process, the researcher had two assistants. The first assistant counted the number of times (frequency) a word, phrase or concept was voted as being important and the second assistant recorded the process in an Excel spreadsheet. The votes are indicated in the frequency column (Table 2). This round with the experts ended when the last item of six or more syllables was voted for and a record of this was kept.

**TABLE 2:** Final list of words, phrases and concepts used for the tool development.

One syllable	Freq	Two syllables	Freq	Three syllables	Freq	Four syllables	Freq	Five syllables	Freq	Six or more syllables	Freq
Salt	17	High blood	13	Blood pressure	13	Check blood pressure	16	Comply with treatment	20	Physical activity	18
Stroke	13	Headaches	12	Stop smoking	12	Silent killer	13	Reduce alcohol	20	Don’t run out of medication	18
Weight	11	Dizzy	7	Lifestyle change	12	Keep appointments	10	Stressful emotions	15	Take medication correctly	18
Pill	10	Walking	7	Tobacco	11	Hypertension	7	High cholesterol	15	Exercise for 30 min	18
Food	8	-	-	Overweight	9	Unhealthy diet	7	Normal blood pressure	14	Don’t borrow medication	10
Drink	7	-	-	Heart failure	8	Obesity	7	Avoid processed food	10	Blocked arteries (coronary artery disease)	6
Oil	6	-	-	Diabetes	6	Avoid fast food	7	-	-	-	-
-	-	-	-	Change diet	6	Vegetables	6	-	-	-	-
-	-	-	-	-	-	Know blood pressure	6	-	-	-	-

Using the record kept by the second assistant in the Excel spreadsheet and starting from one syllable, items were arranged from the highest number of votes to the lowest. Those items that were not voted for were automatically removed from the list. At the end of the meeting, the first panel of experts had selected a number of words according to the syllables (for details, see Table 2).

Round 3 of the modified Delphi technique involved the development of the HHLAT.

The words, phrases and concepts in Table 2 were used in the development of three tools based on a principle similar to the REALM-R tool. In accordance with the REALM-R, the tool has 11 words. Three of these always have one syllable and the other eight have between two and six or more syllables. To decide on the 11 words, phrases and concepts, the words, phrases and concepts with the highest number of votes from the experts (five votes and above) were considered. Words, phrases and concepts with fewer than five votes were not considered at all. For example, the words, phrases and concepts with between one and six syllables with the highest number of votes were considered for the development of Tool 1 (Box 1). The example of the votes is outlined in Table 2.

BOX 1 Tool 1, the Hypertension Health Literacy Assessment Tool.

Patient file number: ____________ School grade completed: ______________

Date of birth: _______/______/____Name of the clinic: _________________

LAB reading level: _________________

Examiner: _________________________

Date of assessment: _______/______/_______

Salt

Stroke

Weight

Headaches________

Blood pressure_____

Stop smoking______

Lifestyle change____

Silent killer_________

Physical activity_____

Comply with treatment___

Reduce alcohol intake____

Salt, stroke, and weight are not scored. A score of 6 or less is used to identify patient at risk for poor hypertension health literacy.

Score: /8

*Source:* Authors’ own work

Tool 1 had as its 11 words, phrases and concepts: salt, stroke, weight, blood pressure, headaches, stop smoking, lifestyle change, silent killer, reduce alcohol intake, physical activity, and comply with treatment. The other tools, Tool 2 and Tool 3, were also developed on a similar principle: the next highest scores were taken and used in the same manner as in Tool 1.

Round 4 involved the validation of the HHLAT.

The validation panel (second panel of experts) was tasked to select and do face validation of one of the three tools developed that would best assess hypertension health literacy in the PHC clinics. Face validity determines whether the newly developed tool ‘looks like’ it is going to measure what it is supposed to measure. The three tools, along with informed consent forms, were sent out through email to the validation panel of 50 experts. They were requested to respond through the same email.

The researcher recorded the responses in an Excel spreadsheet, creating a frequency distribution list. Each time a tool was selected as being most appropriate, it was given a point. This process was given 60 days to allow the participants time to respond. Of the 50 prospective respondents, 30 responded voting Tool 1 (Box 1) as the most appropriate tool to assess hypertension health literacy.

After face validity from the experts, the selected tool was pretested on five participants (patients) in a PHC clinic not to be included in the main study. No adaptations were needed on the tool.

## Data collection and analysis for phase 3: Determining the hypertension health literacy level of patients with hypertension attending primary healthcare clinics in Tshwane, Gauteng province, South Africa

To implement the tool (Table 3), data were collected from 195 patients with hypertension by using the newly developed HHLAT. Field workers who had been trained on how to administer the HHLAT assisted the researcher in this process. The administration and scoring of the tool was similar to the REALM-R tool. The tool consisted of a list of 11 words and phrases. The first three of these items were not counted in the scoring. They were used as icebreakers to relax and calm the participants. The other eight would form the basis for scoring.

**TABLE 3:** Age and gender of the participants (*n* = 195).

Age	Gender		Grand total
	
	Female	Male	
18–25	27	10	37
26–35	51	20	71
36–45	29	19	48
46–55	7	10	17
56–65	6	10	16
66–75	2	2	4
76–85	1	1	2

**Total**	**123**	**72**	**195**

A private, quiet room within the PHC was secured for this purpose. To administer the tool, participants were presented with the tool and asked to pronounce out loud the items as listed. If the participant took more than five seconds on a word, the field worker would say ‘pass’, encouraging the participant to move to the next item. Each field worker had a copy of the list of the words, phrases and concepts, and item pronounced correctly on that list was marked with a tick sign (√), while those pronounced incorrectly were marked with a cross sign (×). If no attempt was made to pronounce the item, a negative sign (-) was assigned. Self-corrected words were marked with a tick sign (√) and counted in the final score. All the tick signs (√) were added up. This score determined the hypertension health literacy level of the participant. Respondents with a score of 6 or lower were considered to be at risk for poor hypertension health literacy.

### Validity and reliability

The REALM-R tool was validated as a shortened version of the REALM and was designed to be used in the public health sector and PHC settings to identify patients with low health literacy levels at the General Internal Medicine Clinic at the University of Kentucky in the year 2000. The REALM-R tool has been shown to correlate with a number of other tests used in health to determine health literacy, such as WRAT-R, TOFHLA, PIAT-R and SORT-R.^12^ The test–retest reliability of the REALM-R tool was 0.99.^13^

In Round 4 of the tool development, the validation panel evaluated the tool to ensure face validity by expressing their opinion on whether they thought the tool would measure what is supposed to measure. For concurrent validity, the HHLAT was administered simultaneously with LAB.

### Ethical consideration

The research was granted ethical clearance by the Tshwane University of Technology (REF: 2013/06/001 [2] [SCI]) and the Gauteng Department of Health (REF: PROJ: 43/2013). All participants in the study were provided with an information leaflet, and they gave consent prior to participation.

## Results

From the validation panel, the number of times an HHLAT was selected as being important and valid was recorded in an Excel spreadsheet and the frequency reflected that Tool 1 scored 63.3% (*n* = 19), Tool 3 scored 23.3% (*n* = 7) and Tool 2 scored 13.3% (*n* = 4). Thus, Tool 1 (Box 1) was used as the HHLAT for determining the hypertension health literacy of patients (*n* = 195) attending selected PHC clinics in Tshwane. Table 3 indicates the age and gender of the participants who took part in the assessment of hypertension health literacy using the newly developed tool.

The 195 participants comprised 123 female and 72 male participants. Their demographic profile revealed that they were aged between 18 and 85 years.

For inclusion criteria, participants self-declared their ability to read English and gave information with regard to their highest school grade, which ranged from never attending school to grade 12. Administering the HHLAT to the 195 participants showed that only 37 (17.7% of women compared to 22.2% men) of the participants were at risk for poor hypertension health literacy. Using Spearman’s rho, we discovered that the risk for poor hypertension health literacy and gender was not significantly associated (*p* = 0.376).

**FIGURE 1:**
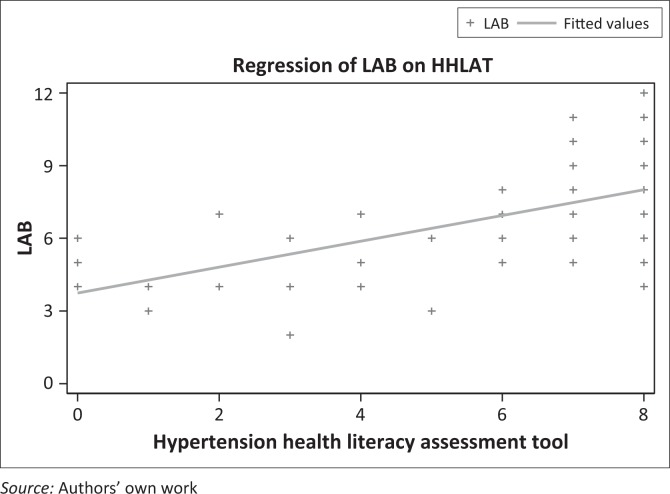
Relationship between Learning Ability Battery (LAB) and Hypertension Health Literacy Assessment (HHLAT).

Administration of the tool took less than two minutes per participant, which is similar to the duration of the REALM-R tool. For the purpose of validity, the HHLAT was administered concurrently with the LAB for concurrent validity, and a strong positive correlation was observed (Figure 1). Figure 1 illustrates the relationship between LAB and HHLAT.

## Discussion

The Delphi technique has been used by other authors^14,15^ for examining health promotion and health education where it was found suitable. The modified Delphi technique used in this study comprised a first panel of experts and a second panel of experts (validation panel). The first panel of experts reached a conclusion within one day, as they met in one room. During this meeting, a list of important common words or phrases and concepts used during hypertension health education was created and listed according to those who were voted to be most important to the least. This list can be used by other researchers in health promotion or education. During the process of the tool development, no list similar to the one that has been created in this study existed. The second panel of experts (validation) participated by means of email and they were given 60 days to respond by selecting and validating the most appropriate HHLAT for PHC clinics from the three tools that were developed. This process prevented bias as each participant did not know who was sent an email and their responses were not influenced by other participants.

Men (72) represented a quarter of the total sample and posed a higher risk (22.2%) of being at risk for poor hypertension health literacy. For those who were at risk, it could be attributed to several factors such as gender and basic schooling and health-seeking behaviours. In their study in 2014, Gibbs, Sikweyiya and Jewkes^16^ revealed that most men in South Africa left school early and did not complete high school. Also, men are known to only use health clinics when they are forced by circumstance but not for general check-ups as compared to women.^17^ Thus, men miss out on the opportunity to receive all that healthcare clinics have to offer, including hypertension health education. Because of their reluctance to visit PHC clinics, men may not be familiar with the words, phrases and concepts commonly used during hypertension health education. Significantly in this study, a small (17.7%) proportion of women (*n* = 123) were at risk for poor hypertension health literacy, and taking these results into consideration might be an indication of the literacy and health literacy status of women using the four PHC clinics. Furthermore, evidence from the STATS SA report revealed that 92% of South Africans are able to read and write.^18^ Another key point to remember is that this may suggest a positive move towards women empowerment for South Africa, especially because globally women were disadvantaged when it comes to education.

With regard to hypertension health literacy, the results showed that less than a fifth (19%) of the participants had poor hypertension health literacy. Health literacy is the key outcome of health education and is divided into three distinct levels.^19^ The first level is functional literacy, where the individual is able to basically read and write at a level that is necessary for effective functioning in the health system. The second level is interactive level, which involves the more advanced cognitive literacy and social skills that enable active participation in the healthcare system. The third level is where the patient is able to critically analyse and use information to participate in actions that overcome structural barriers in health.^9^ The HHLAT tool assessed hypertension health literacy at the first level of health literacy. Poor health literacy is associated with poor health status, and those with poor health literacy are at risk for frequently using emergency rooms, missing hospital appointments and having a record of poor compliance.^20^ This is evident as seen in a study by Hanan and colleagues, which revealed that patients with hypertension and with low health literacy asked fewer questions than those with adequate literacy.^21^ In a study conducted in German primary healthcare, researchers discovered that patients with hypertension and adequate health literacy participate in decision-making and were likely to be satisfied with the care they received from the clinics.^22^ Miranda and co-authors conducted a study which revealed that Ghanaian participants with low health literacy were less likely to achieve blood pressure control than Ghanaians with adequate health literacy. The authors further emphasised that efforts to improve health literacy may reduce prevalence and improve awareness and control of hypertension.^23^

Adequate health literacy is necessary for patients with hypertension as they are required to comply with treatment; they need to read medications as prescribed by the healthcare provider, follow instructions when using the medication and read food labels when buying groceries for non-pharmacological management of hypertension. With poor health literacy or at risk for poor health literacy, a patient might overlook important information that is presented in a written form, leading to non-compliance.^13^ The significance of literacy and health literacy in patients with chronic diseases such as hypertension cannot be over-emphasised because these patients will take care of themselves for a lifetime. Therefore, they need adequate health literacy to do so, as was also mentioned by Dube et al.^24^ To ensure validity, the HHLAT was administered concurrently with the LAB.

The association between the HHLAT and the LAB was confirmed by fitting a linear regression model of LAB on the HHLAT. There was overwhelming positive evidence that LAB increased with increasing HHLAT (*F* = 76.0, *p* < 0.0001, *R*^2^ = 28.25%). This means that the HHLAT can be regarded as valid.

## Conclusion

Using the newly developed HHLAT, the hypertension health literacy levels of patients in PHC clinics can now be determined. This tool can be administered by a healthcare worker in less than two minutes, without disruption of the normal functioning of the clinic. Determining the hypertension health literacy levels of patients will enable the healthcare provider to specifically individualise their health education. When health education is planned and provided to the public, it needs to be simple and easy to comprehend in order for learning to take place. This is because the tool revealed that only 19% of the participants were at risk for poor hypertension health literacy. Further research is required to investigate the compliance to pharmacological and non-pharmacological management related to hypertension as these findings indicate that most (81%) of the participants are hypertension health literate, while the country is experiencing poor control of hypertension. What still needs to be further explored is whether they use this knowledge to live a healthy lifestyle and comply with the management of hypertension.
